# PReS13-SPK-1030: Juvenile dermatomyositis

**DOI:** 10.1186/1546-0096-11-S2-I18

**Published:** 2013-12-05

**Authors:** P Quartier

**Affiliations:** 1 Pediatric Immuno-Haematology and Rheumatology Unit, Necker-Enfants Malades, Paris, France

## 

Juvenile Dermatomyositis (JDM) is a systemic, inflammatory, idiopathic disease, mainly affecting the skin and the muscles, starting before the age of 16 with an incidence around one case per million children. Some patients display typical features of JDM without skin involvement or even without muscle involvement, however both tissues are affected over time in most cases. Diagnosis criteria have been established by Bohan and Peter 35 years ago, based on the presence of typical skin rash and proximal muscle involvement. Other conditions have to be rulled out before making a diagnosis of JDM, such us other connective tissue diseases, polymyositis, infectious/post-infectious myositis, genetic diseases, metabolic or drug-induced myopathies. Unlike adult-onset dermatomyositis, JDM is exceptionnaly associated with a malignant disease.

JDM may also affect several organs including the lungs and the digestive tract. In a subset of patients, glucose intolerance, lipodystrophia and/or calcinosis develop. Delay in treatment initiation or inadequate treatment may favour diffuse, debilitating calcinosis.

JDM patients have to be referred to reference pediatric centers with a multidisciplinary team to properly assess disease activity, disease-related damage (including low bone density in most cases) and define the best treatment. Long-lasting corticosteroid therapy remains the gold standard, together with physiotherapy. Physiotherapists often play an important rule not only through their participation to patients care but also by assessing disease activity at each stage, using several tools including CMAS and MMT assessment of muscle strength. Other tools have been developed to assess extra-muscular disease and disease-related damage. Patients quality of life also needs to be properly assessed. Several collaborative efforts have been conducted to improve the way we assess patients health and response to treatment.

Most patient respond to treatment, relapses are frequent but a complete disease remission is achieved in most cases before adulthood, with or without sequellae. Ongoing clinical trials are assessing the effect of several immunosuppressive and immunomodulatory drugs; preliminary results from a PRINTO international trial suggest that both methotrexate and cyclosporine may help controlling the disease, reducing the rate of early flares and may hence have a corticosteroid-sparing effect. On the short term, methotrexate seems better tolerated. Some preliminary data suggest that other drugs may also be of interest.

## Disclosure of interest

None declared.

